# Patient Controlled, Off-label Use of Continuous Glucose Monitoring: Real-World Medical Costs and Effects of Patient Controlled Sensor Augmented Pump Therapy in Adult Patients Type 1 Diabetes

**DOI:** 10.1177/1932296820920909

**Published:** 2020-06-09

**Authors:** Patrik Hidefjäll, Lars Berg

**Affiliations:** 1 Unit for Bioentrepreneurship, Karolinska Institutet, Stockholm, Sweden; 2 Diabetes Nurse, Medical Clinic, SÄS Borås, Sweden

**Keywords:** continuous glucose monitoring, cost analysis, effect analysis, Sweden, adults, type 1 diabetes, off-label use

## Abstract

**Background:**

Continuous glucose monitoring (CGM) has shown promise to reduce glycated hemoglobin (HbA1c) levels, but its cost-effectiveness is seen as uncertain by reimbursement agencies. The aim of this study was to explore the impact of real-world, off-label, patient controlled CGM use in combination with continuous subcutaneous insulin infusion (CSII) on costs and effects in patients with type 1 diabetes in a Swedish clinic.

**Methods:**

A real-world, retrospective study with questionnaire on CGM use by adult patients with type 1 diabetes on CSII (Animas Vibe) were offered sensor augmented pump therapy (SAPT) (Dexcom G4) as part of hospital innovation funding program. Direct medical costs, HbA1c, and complications following switch from CSII with self-monitoring of blood glucose (SMBG) to SAPT were calculated.

**Results:**

Questionnaire data showed that CGM sensors were on average used 92% of the time for 22 days. One hundred and thirty-nine (95%) of 146 respondents used each sensor for longer than one week. Data analysis showed a statistically significant HbA1c decrease of 0.56% (6.1 mmol/mol) after change to SAPT. In patients using the sensor 100%, the decrease was 0.89% (9.8 mmol/mol). The analysis showed that SAPT led to higher costs (5500 USD/year) than CSII + SMBG (3680 USD/year), with incremental costs being 1815 USD per year to achieve an HbA1c decrease of 0.56% (6.1 mmol/mol). The incidence of all complications declined after switch to SAPT.

**Conclusion:**

The primary data analysis showed a decrease in HbA1c values following switch to SAPT, corresponding to previous cost-effectiveness studies, but at substantially lower costs due to longer sensor off-label use.

## Introduction

Continuous glucose monitoring (CGM) was commercialized 20 years ago,^1^ but has only recently gained importance in Swedish clinical practice.^2^ A main reason that CGM has not been adopted quicker is most likely due to its costs and uncertain cost-effectiveness, compared with self-monitoring of blood glucose (SMBG) in patients with type 1 diabetes.^3,4^ There is consequently a need for additional real-world cost-effectiveness analyses of use of CGM in type 1 diabetes.^4^ To avoid methodological problems of cost-utility studies,^5,6^ a feasible alternative could be to focus on established outcomes such as glycated hemoglobin (HbA1c), complication rates, and the direct medical costs associated with CGM use. This would include costs of acute complications such as severe hypoglycemia, hyperglycemia, and ketoacidosis.

This suggested approach would also make effects of sensor use practice on HbA1c, complications, and costs more obvious. The first versions of sensors were intended for use up to 72 hours, while sensors to date are labeled for 10 days without need for calibration.^7^ This suggests sensor duration will be longer than today, resulting in reduced costs per hour of use.

A common practice in medicine is user-initiated innovation to satisfy unmet clinical needs by off-label use of given technology.^8^ Off-label use means when a product is used in another way than intended by the manufacturer. Sharing experience with off-label use helps practitioners consider involved trade-offs, but also requires clinicians to report adverse events and provide transparency and autonomy to patients.^9^ In this study, patients were informed of the possibility to use their CGM sensors longer than the stated duration in the instructions for use. As longer use of a CGM sensor reduces costs, it offers the possibility to treat additional patients. This was an important driver for the patients in the described study, who out of solidarity were helping their fellow patients also gain access to CGM and sensor augmented pump therapy (SAPT) from the fixed diabetes clinic budget. Additionally, if accumulating experience shows that longer sensor use is feasible, it may also lead to updated labeling and reimbursement agencies considering CGM cost-effective. Consequently, the aim of this study was to explore in a real-world setting the impact of patient controlled, off-label CGM use on costs and effects.

## Research Design and Methods

### Study Design, Patient Population, and Devices Used

One hundred and eighty-eight adult patients at the diabetes clinic in Borås using continuous subcutaneous insulin infusion (CSII) (Animas Vibe) were, from October 2011 and onward, offered to add a compatible CGM system (Dexcom G4) to receive SAPT. Extra funding for introduction of new methods (hospital innovation fund) had been assigned for two years to the diabetes clinic to introduce SAPT. Only patients who were deemed capable of managing the SAPT system appropriately were offered this opportunity. Patients were instructed on how to reset the sensor, to use their CGM as long as it worked accurately and to do blood glucose tests at least two times per day to calibrate the sensor. Furthermore, they were instructed to pay attention to skin irritation and replace the sensor as soon as symptoms occurred. As patients were starting SAPT at various calendar dates, retrospective outcome data could be collected according to a before-after study design. Data of these patients (HbA1c lab values at regular follow-ups and other blood values [pH value, salt balance, blood glucose levels, etc.] at visits to emergency room, hospital with or without ambulance) were obtained from electronic patient records. The number of complications that required acute health care assistance was included based on available entries in the record from visits to the emergency unit, ambulance calls, or inpatient visits. All health care contacts were analyzed as to whether they had been primarily diabetes related, based on the main diagnosis or laboratory values.

Data from patients’ electronic medical records were entered manually into Excel in February and September 2014 and April 2015, covering the time span from October 2010 until April 2015, that is, data before and after the start of SAPT. One patient had not yet in April 2015 started SAPT, consequently leading to 188 patients before and 187 patients after start of SAPT. The amount of available data varied between the patients according to their level of contacts with health care.

A questionnaire, containing 10 closed questions with 5-7 alternatives per question and 1 open question, was sent out in October 2014 by mail to all 188 patients. Questions related to CGM sensor use (importance and benefit of, frequency and period of sensor use, calibration frequency), daily blood glucose measurements when CGM is not used, use period of infusion set, and frequency and mode of data reporting to the diabetes clinic.

### Statistical Analysis

The main effect analyzed in this study was the change in HbA1c before and after the start of CGM use based on 188 available datasets. The data analysis was done in Excel and SPSS, with systematic cross-checks to ensure data accuracy. The change in HbA1c was calculated for the combined dataset (from October 2010 until April 2015) before and after the start of SAPT, using mean values per patient and period to ensure that each patient had the same weight of the average, irrespective of the number of recorded values. The difference in mean HbA1c values was tested for statistical significance (*P* < .05) using the paired *t*-test in SPSS.

All HbA1c values of each patient were classified according to their occurrence either within one or two years before or after the individual dates of switch to SAPT to identify their temporal development. SAPT was offered, with an option of off-label use from end of October 2011 resulting in 187 patients ultimately using SAPT and one patient not yet starting SAPT. Four patients changed from SAPT to insulin-pen and one patient moved from Borås after some CGM data had been recorded. As patients switched to SAPT at different calendar dates, patients were seen at different frequency, the number of patients included and HbA1c values recorded in the four different periods differed. There were on average 4.8 (42%) recordings per patient before and 6.7 (58%) recordings after the switch to SAPT. The number of patient-months before switch to SAPT was 4 310 (43%) and after switch to SAPT was 5 645 (57%), indicating no change in the follow-up scheme of patients.

To assess the impact of sensor use on HbA1c levels, patients were divided into five groups: “100% sensor use” (*n* = 55), “At least 90% sensor use” (*n* = 119), all questionnaire responding patients (*n* = 146), questionnaire nonresponding patients (*n* = 42), and finally all 188 patients. The frequency of CGM use was calculated by combining the averages of the given interval for length of use and time it takes until the next sensor is started. A difference in mean HbA1c reduction between questionnaire responding and nonresponding patients was tested for its statistical significance (*P* < .05) using the *t*-test in SPSS.

The number of complications occurring within the observed time periods before and after switch to SAPT were divided by the respective time periods in patient-months before (4 310) and after (5 645) switch to SAPT.

The annual costs per patient were analyzed for two years before and after starting SAPT. All direct costs related to the treatment of type 1 diabetes were included. Costs of device use came from tenders of Västra Götaland Region.^10^ Costs for CGM training and health care contacts were based on available standard costs. Costs were translated to the average historical exchange rate of 6.7850 SEK/USD of the period studied.^11^

The amount of material (CGM sensors, transmitters, test strips, and infusion sets) used per year was taken from the questionnaire data using average values of each interval alternative. Actual CGM sensor consumption over a period of 10 months for the whole clinic was used as a plausibility check. Costs were allocated to the year of their occurrence with investments divided over their remaining use period.

### Ethical Considerations

Off-label use of CGM sensors was not initiated as part of any planned study and only retrospectively documented in this study. All patients were instructed to check their blood glucose levels before meals or at least twice daily to make sure the CGM sensor was correctly calibrated and to stop use the sensor when calibration no longer was possible to avoid adverse events, irrespective of actual duration of sensor use.

An ethics committee approval was granted (ref. 2014/112-31/1) before patient data were collected. Data were anonymized by the clinically practicing author, giving each patient an identity number and keeping the correlation key safe.

## Results

As the dataset was taken from the patients’ medical records, it represents real-world clinical practice. This is reflected in that not all the patients switched to SAPT at the same point in time and therefore there are different amounts of recorded HbA1c values available for each patient. The background characteristics of the study population can be found in Table 1.

**Table 1.** Background Characteristics of the Study Population (April 2015).

Variables	Values
Females—no. (%)	101 (54)
Mean age—years (range)	36 (18-77)
Mean weight—kg (range)	77 (47-127)
Mean BMI (range)	26 (18-38)
Mean diabetes duration—years (range)	23 (2-59)
Mean daily insulin units (range)	45 (16-125)
Tobacco use—no. (%)	
Nonsmokers	157 (84)
Cigarettes and snuff (powder tobacco)	31 (16)
Alcohol use—no. (%)	
No or seldom	97 (52)
Moderate	32 (17)
Regular	33 (18)
Physical activity—no. (%)	
<1 time/wk	12 (6)
1-2 times/wk	16 (9)
>3 times/wk	160 (85)
Mean systolic blood pressure (range)	121 (165-95)
Mean diastolic blood pressure (range)	72 (110-59)
Eye status—before no. (%)/after no. (%)	
No changes or simplex retinopathy	155 (84)/145 (79)
Worse than simplex retinopathy	30 (16)/39 (21)

Abbreviation: BMI, body mass index.

The results of the effect analysis are displayed in Table 2. The analysis of the whole dataset showed that there was a decrease in HbA1c of 0.56% (6.1 mmol/mol), which was statistically significant (*P* < .05). The statistical significance of the change in HbA1c was analyzed based on the difference in HbA1c values from before and after the start of SAPT for each individual patient. Of all 188 patients, 157 had a lower mean HbA1c value while 31 patients had a higher mean HbA1c value following use of SAPT. The temporal development of the HbA1c values showed that the mean values for the whole dataset dropped observably following the introduction of SAPT.

**Table 2.** Mean HbA1c Values (in %/mmol/mol (SD)(*n*)) for the Study Population by Analysis (April 2015).

Overall	Before Sensor-Use/SAPT 8.4/67.4 (12.2) (188)		During SAPT	Difference
			7.8/61.3 (13.0) (187)	−0.56/ −6.1 (8.8) (187)
**In % sensor-use**	**100%**	**>90%**	**All reported**	**Not reported**
**During SAPT**	7.3/56.8 (10.1) (55)	7.5/58.4 (9.7) (119)	7.5/58.9 (9.7) (146)	8.5/69.9 (18.5) (42)
Reduction	0.89/9.8 (6.8) (55)	0.72/7.8 (7.3) (119)	0.63/6.9 (7.8) (146)	0.29/3.1 (11.2) (42)
**Sensor-use length**	**1-2 mo**	**2-4 wk**	**1-2 wk**	**1 wk or less**
Reduction	0.96/10.5 (8.5) (29)	0.50/5.5 (7.7) (79)	0.75/8.2 (6.5) (31)	0.25/2.7 (2.6) (7)
**Pause of sensors**	<**1 h**	<**24 h**	**1-3 d**	>**4 d**
Reduction	9.9/0.91 (6.8) (52)	6.6/0.61 (7.5) (53)	4.7/0.43 (5.4) (17)	2.1/0.19 (9.2) (21)
**Development**	**2 y before**	**1 y before**	**1 y after**	**2 y after**
	8.3/67.2 (12.0) (138)	8.3/67.4 (12.8) (185)	7.9/ 62.5 (13.1) (183)	7.8/61.4 (14.1) (170)

Abbreviations: BMI, body mass index; HbA1c, glycated hemoglobin; SAPT, sensor augmented pump therapy; SD, standard deviation.

When studying the mean HbA1c values in dependence of the frequency of sensor use, a larger decrease in HbA1c could be observed for the 55 patients that used the sensor 100% of the time, that is, 0.9% (9.8 mmol/mol) down to 7.3% (56.8 mmol/mol). Also the 119 patients that used the sensor 90% or more and all 146 patients that reported sensor use in the questionnaire had HbA1c reductions of 0.72% (7.8 mmol/mol) and 0.63% (6.9 mmol/mol), respectively. The group of 42 patients (22%) that did not answer the questionnaire had a significantly (*P* < .05) higher HbA1c of 8.5% (69.9 mmol/mol) compared with the whole patient group following the switch to SAPT. Also the reduction in HbA1c of 0.29% (3.1 mmol/mol) was significantly lower (*P* < .05) for those not answering the questionnaire compared to those who answered it 0.63% (6.9 mmol/mol). The mean reduction of HbA1c was larger in patients who used sensors longer and had shorter interruptions between sensors.

One hundred and forty-six of 188 (78%) patients answered the questionnaire, which first seven questions are provided in Table 3, with a result that showed the high importance sensor use had for these patients to manage their diabetes. The results of sensor use confirmed the calculation based on total sensor consumption in the clinic and the impression by the clinically responsible nurse that patients felt confident in using the sensors longer than the intended seven days. Average sensor duration was 22 days for the respondent population. As interruptions between sensors were short, the average calculated sensor use frequency was 92%.

**Table 3.** First Seven Questions and Results of the Questionnaire.

Question 1: What is the importance of the CGM sensor for your ability to adjust your insulin does?	n	%
Very high importance	115	78
High importance	28	19
Moderate importance	4	3
Low importance	0	0
Very low importance	0	0
Question 2: What benefits do you experience of the CGM sensor?	*n*	%
Provides confidence of avoiding hypoglycemia	128	21
Provides confidence of avoiding hyperglycemia	127	20
Makes it easier to avoid large blood sugar variations	125	20
Helps me gain knowledge how to adjust my insulin doses	133	21
Helps me gain knowledge about how to adjust my behavior to improve health	109	18
I don’t see any direct gains from use of the sensor	0	0
Question 3: How often do you use the CGM sensor?	*n*	%
As often as possible with change to a new sensor within one hour	52	36
As often as possible with change to a new sensor when suitable within 24 h	53	36
Regularly with some interruption (1-3 d) between the used and the new sensor	17	12
Periodically with interruptions (4-7 d) between the used and the new sensor	11	8
Sporadically with longer interruptions (several weeks) between the used and the new sensor	10	7
I have stopped using the CGM sensor	3	2
Question 4: For how long do you usually use the CGM sensor?	*n*	%
For 1 to 2 mo until the sensor no longer works	29	20
For 2 to 4 wk until the sensor no longer works	79	54
For 1 to 2 wk until the sensor no longer works	31	21
For approximately one week	5	3
For less than one week	2	1
Question 5: How often do you calibrate the CGM sensor per day?	*n*	%
Five or more times per day	1	1
Four times per day	10	7
Three times per day	21	14
Two times per day	80	55
One time per day	21	14
Less than one time per day	13	9
Question 6: During breaks when not using the CGM, how often do you measure blood glucose values with test strips per day?	*n*	%
Five or more times per day	83	57
Four times per day	23	16
Three times per day	13	9
Two times per day	15	10
One time per day	5	3
Less than one time per day	6	4
Question 7: How many days do you normally use each infusion set for your insulin pump?	*n*	%
Seven days or longer	4	3
5-6 d	20	14
3-4 d	42	30
Approximately 3 d	48	34
2-3 d	25	18
1-2 d	3	2

Abbreviation: CGM, continuous glucose monitoring.

The analysis of health care contacts showed that the incidence of complications was low with 2.5 hypoglycemia, 7.0 hyperglycemia, and 1.4 ketoacidosis events per 100 patient-years before switch to SAPT and 1.7 hypoglycemia, 3.6 hyperglycemia, and 1.3 ketoacidosis events per 100 patient-years after switch to SAPT. A detailed analysis of the individual events revealed some patients having multiple incidents, which were deemed to be due to patient misjudgments of the needed insulin doses, not due to patient hypo-unawareness or CGM sensor inaccuracy. Regular follow-up visits took place two to three times a year per patient.

The annual direct medical costs were calculated per patient based on mean values from the whole study population. It can be seen that the costs for emergency room visits, inpatient visits, ambulance contacts, and intensive care unit visits due to complications increased from two years to one year before the start of SAPT and then decreased in the first and even more in the second year after starting SAPT. The analysis showed that SAPT led to higher costs (5500 USD/year) than CSII + SMBG (3680 USD/year), with incremental costs being 1815 USD per year. All costs (USD) are displayed in Table 4.

**Table 4.** Annual Average Direct Medical Costs per Patient in USD.

	2 y before	1 y before	1 y after	2 y after
CSII	995	995	995	995
Transmitter (CGM)	0	0	688	688
**Investment goods total:**	995	995	1683	1683
Reservoir	302	302	302	302
Infusion tube	1306	1306	1306	1306
Test strips	356	356	158	158
Lancets	11	11	11	11
Insulin	418	418	418	418
Batteries	53	53	53	53
Reservoir lid	16	16	16	16
Battery lid	32	32	32	32
Sensor (CGM)	0	0	1134	1134
**Consumables total:**	2495	2495	3431	3431
CGM training	0	0	442	0
Regular follow-up	64	126	132	89
Emergency room	6	22	22	6
Inpatient visit	0	51	28	5
Intensive care unit	0	62	0	0
Ambulance	13	39	33	10
**Medical services total:**	83	301	657	111
**Sum**	3573	3791	5771	5224

Abbreviations: CGM, continuous glucose monitoring; CSII, continuous subcutaneous insulin infusion.

## Discussion

The analysis of the clinical data showed that the decrease in mean HbA1c of 0.56% (6.1 mmol/mol) of using SAPT compared with CSII + SMBG was statistically significant (*P* < .05). Also, the temporal development of HbA1c showed higher HbA1c values before switch to SAPT, dropping significantly afterwards and remained lower during the second year after switching to SAPT. The decrease in HbA1c values was also larger when the CGM sensor was used 100% of the time. Patients who used the sensors longer and had shorter interruptions between each sensor had larger reductions in their HbA1c values. However, as the research design did not use an independent control group we should avoid drawing strong conclusions that this difference in HbA1c was caused by sensor use or sensor use duration. Also the educational effort in the intervention most likely contributed to better results. There was also a lower incidence of complications per patient years after switch to SAPT compared to before, despite using sensors longer than its intended use (off-label), thus indicating that off-label use as practiced in this study was safe. A limitation in our data is that only complications leading to documented health care contacts were captured and that mental health data or the existence of hypo-unawareness was not captured in the description of the studied cohort (Table 1). On the other hand, this adult patient group had been followed clinically for many years by one of the authors (LB) and was judged capable of handling the SAPT system, also in an off-label fashion, if the patients chose to do so.

The questionnaire response rate of 78% was satisfactory, given that the questionnaire required the effort of sending it back by postal mail. As the patient population of this study represented almost all patients in the clinic on CSII, we have a broader group of patients than only those with unstable glycemic control and recurrent hypoglycemia. Despite this broader patient population there is an overwhelmingly clear benefit of CGM sensor use, as reported in the questionnaire. However, the 42 patients who did not answer the questionnaire had a significantly (*P* < .05) higher HbA1c after start of sensor use and significantly (*P* < .05) lower HbA1c decrease compared to those 146 patients who answered the questionnaire, possibly indicating a lower engagement in their therapy.

As previous cost-effectiveness studies relied on the difference in HbA1c between CGM and SMBG (with or without CSII) to calculate a difference in Quality of Life, based on the Diabetes Control and Complications Trial, the achieved HbA1c difference is of importance for the overall cost-effectiveness of CGM.^12-16^ As three of the four cost-effectiveness studies were conducted in the United States, they may not be appropriate comparisons to CGM costs in Sweden. Despite this we can observe that the annual direct medical costs reported in the US cost-effectiveness studies were on average roughly twice the cost of the 5497 USD found for SAPT in this study. Also the sensor specific costs of 1822 USD of this study were substantially higher in the US studies with 4335 USD,^13^ 4189 USD,^14^ and 3343 USD.^15^ If we instead compare our annual costs for CGM of 1815 USD with previously reported CGM specific costs in Sweden by Statens Beredning för Medicinsk och Social Utvärdering (Swedish government agency for health technology assessment) (SBU) at 4371 USD^17^ and the study by Roze et al,^16^ in which direct annual sensor costs were 3593 USD based on 48 sensors and one transmitter (Minilink + Serter), we can note that our reported costs are substantially lower, also compared with other studies in a Swedish setting. The difference is mainly due to the longer off-label sensor use of 22 days (15,3 sensors annually), whereas the frequency of sensor change was assumed to be 7 days by SBU^17^ and slightly longer by Roze et al.^16^

If we speculate about the cost-effectiveness implications of this study relative to the four previously discussed cost-effectiveness studies, we can determine that the 9% relative reduction of HbA1c of 0.56% (6.1 mmol/mol) is both clinically relevant and comparable to other studies, but at roughly half the direct medical costs, even compared to studies in a Swedish setting. The substantially lower costs are mainly due to the less frequent change of the CGM sensor, of every three weeks (22 days) instead of every seven days. On the effectiveness side, we could have tried to include quality of life aspects which also, based on the questionnaire data, indicate substantial gains.^18^ This leads us to discuss the special conditions of this study and the off-label use of CGM sensors.

A major limitation of this study is that the sensor and insulin pump technologies used in this study (Animas Vibe and Dexcom G4) are now at this study’s publication (2020) superseded and that newer technologies may not offer the same off-label potential. Also, the study reflects Swedish practice during the time 2010 to 2015, which means that other off-label practices, later available is not covered, or discussed. The retrospective study design and lack of control group does not allow conclusions to be drawn about the causality of the relationship between the use of CGM and the reduction in HbA1c. However, the difference in mean was statistically significant. Based on the analysis of the HbA1c effect with regard to the frequency, duration, and intermittency of sensor use, trends between a more frequent, longer, and less intermittent CGM sensor use and a lower mean HbA1c value were identified. Also, the level of complications following the switch to CGM also declined for severe hypoglycemia, hyperglycemia, and ketoacidosis.

Based on the demonstrated feasibility of using CGM sensors longer than its intended use in this study and the introduction of sensors by other manufacturers with longer duration, we expect a general trend of CGM labeling for longer term use. If that happens and unit prices are kept the same, future cost-effectiveness studies will likely show CGM to be cost-effective. This will likely pave the way for broader use of CGM sensors in type 1 diabetes patients.

## Conclusion

The results of the study showed a statistically significant and clinically relevant decrease in HbA1c of 0.56% (6.1 mmol/mol) comparing CSII + SMBG to SAPT. In patients using CGM sensors 100% of the time the reduction in HbA1c was even larger, with 0.9% (9.8 mmol/mol). Higher CGM use led to lower HbA1c levels, even when using sensors longer than their intended use. During the extended use of CGM sensors, the level of complications declined following use of CGM and no major adverse events due to sensor functionality were reported, indicating that off-label use of the specific CGM sensor as practiced in this study’s setting was safe.

The costs for SAPT in the first year following switch to CGM sensors used 92% of the time were 5771 USD and in the subsequent year 5224 USD. Costs for use of CSII + SMBG were 3573 USD two years before and 3791 USD one year before switching to SAP. The mean additional cost of SAPT compared to CSII + SMBG therefore was 1815 USD per year to reduce HbA1c levels by 0.56% (6.1 mmol/mol), which is substantially lower than other comparable cost-effectiveness studies and due to off-label sensor use. This leads us to expect CGM sensors labeled for longer use with improved cost-effectiveness.
